# DFT Calculation and MD Simulation Studies on Gemini Surfactant Corrosion Inhibitor in Acetic Acid Media

**DOI:** 10.3390/polym15092155

**Published:** 2023-04-30

**Authors:** Mohd Sofi Numin, Khairulazhar Jumbri, Kok Eng Kee, Almila Hassan, Noorazlenawati Borhan, Juan Matmin

**Affiliations:** 1 Department of Fundamental and Applied Sciences, Universiti Teknologi Petronas, Seri Iskandar 32610, Malaysia; 2 Department of Mechanical Engineering, Universiti Teknologi Petronas, Seri Iskandar 32610, Malaysia; 3 Petronas Research Sdn Bhd (PRSB), Jalan Ayer Hitam, Bandar Baru Bangi 43000, Malaysia; 4 Department of Chemistry, Faculty of Science, Universiti Teknologi Malaysia, Johor Bahru 81310, Malaysia

**Keywords:** Gemini surfactant, corrosion inhibitor, MD simulation, DFT calculation, adsorption

## Abstract

Gemini surfactant corrosion inhibitor (CI) is one type of CI mainly used in mitigating corrosion in the complex system of oil/gas production industries. Computer modeling methods such as density functional theory (DFT) calculation and molecular dynamic (MD) simulation are required to develop new CI molecules focusing on their application condition as a prediction or screening process before the physical empirical assessment. In this work, the adsorption inhibition efficiencies of two monomer surfactants (2B and H) and their respective Gemini structures with the addition of different spacers (alkyl, benzene, ester, ether, and ketone) are investigated using DFT calculation and MD simulation method in 3% sodium chloride (NaCl), and 1500 ppm acetic acid solutions. In DFT calculation, 2B-benzene molecules are assumed to have the most promising inhibition efficiency based on their high reactivity and electron-donating ability at their electron-rich benzene ring region based on the lowest bandgap energy (0.765 eV) and highest HOMO energy value (−2.879 eV), respectively. DFT calculation results correlate with the adsorption energy calculated from MD simulation, where 2B-benzene is also assumed to work better as a CI molecule with the most adsorption strength towards Fe (110) metal with the highest negative adsorption energy value (−1837.33 kJ/mol at temperature 323 K). Further, diffusion coefficient and molecular aggregation analysis in different CI concentrations through MD simulation reveals that only a small amount of Gemini surfactant CI is needed in the inhibition application compared to its respective monomer. Computer simulation methods successfully predict and screen the Gemini surfactant CI molecules that can work better as a corrosion inhibitor in acetic acid media. The amount of Gemini surfactant CI that needs to be used is also predicted. The future planning or way forward from this study will be the development of the most promising Gemini surfactant CI based on the results from DFT calculation and MD simulations.

## 1. Introduction

The oil and gas industrial equipment damage is mainly caused by the aqueous environment that can promote corrosion, which can occur in various conditions of oil and gas production, processing, and pipelines system [1]. The presence of high-impurity products in crude oil and natural gas induces the production of corrosion particles such as dissolved hydrogen sulfide (H_2_S), chlorine (Cl_2_), oxygen (O_2_), and carbon dioxide gaseous. The continual production of these corrosion particles will form an internal surface at the material’s components, severely affecting the materials. The components exposed to these corrosion particles will undergo materials degradations due to the various operating conditions in oil and gas production that require extreme temperature and pressure. The materials’ degradation can lead to thickness reduction, loss of materials, ductility, and strength reduction, where the worst case scenario is that the component is completely broken down, needs to be replaced, and production needs to be stopped [2]. Mild steel is the construction material commonly used in this equipment production, such as pipelines, flooding systems, storage tanks, and heat exchange [3]. The corrosion on the mild steel surface causes enormous economic losses and environmental risks and threatens human life. In 2013, NACE reported that the global cost of corrosion is estimated to be USD 2.5 trillion, equivalent to 3.4% of the global gross domestic product (GDP) [4]. The same report also stated that 15 to 35% of the cost of corrosion could be saved using the available corrosion control particles.

Corrosion can be controlled by hindering the natural chemical reactions between the metal and its environment. The common corrosion control particles used in the oil and gas industry are corrosion-resistant alloys (CRAs), protective coatings, corrosion inhibitors (CI), and cathodic protection. Of all the corrosion control strategies, corrosion inhibitors are highly considered an economical and effective way of mitigating internal corrosion [5]. In general terms, a corrosion inhibitor can be defined as a substance that, when added in a small concentration to an environment, will effectively reduce the corrosion rate of the metal exposed to the environment [6,7,8]. Gemini surfactant is one corrosion inhibitor that is famously studied by researchers nowadays in acidic and seawater environments due to its unique properties that can contribute to its characteristic in preventing corrosion [9]. In 2011, Mahdavian et al. (2011) found a cationic Gemini surfactant molecule, namely, N, N′-didodecyl-N,N,N′,N′-tetramethyl-1,4-butanediammonium dibromide (12-4-12) has worked better as a corrosion inhibitor with a low corrosion rate compared to its monomeric surfactant dodecyl trimethylammonium bromide [10]. Other than that, Zhou et al. (2019) also compared the imidazolium surfactant [C14-4-C14im] Br_2_ and its corresponding monomer [C14mim] Br CIs for A3 carbon steel in HCl solutions and found that the Gemini surfactant gave a better inhibition efficiency value. On the other hand, they also found the importance of adsorption strength that can affect the CI performance, where the [C14-4-C14im] Br_2_ Gemini CI surfactant gave a three times adsorption entropy value higher than its corresponding monomer [C14mim] Br [11].

Adsorption is the critical aspect for the inhibition activities of the surfactant molecule towards the metal surface. The adsorption properties of the surfactant CI molecules towards the metal surface and the production of a protective layer influence the efficiency of CI in impeding corrosion processes [12]. The hydrophilic parts of the surfactant molecules that consist of several heteroatoms (N, O, P, S) will be the key for the adsorption processes to occur due to the presence of extra electrons that can be donated to the metal atoms’ empty (3d orbitals) on the metal surface. Having two hydrophilic parts and a spacer in the Gemini surfactant structures may increase the adsorption strength towards the metal surface by two-fold more than the monomer ones. These can be confirmed through an in-depth study using computational methods to study the adsorption properties of the Gemini surfactant and its conventional monomer towards the metal surface. It will also give a better explanation on the inhibition mechanism of the corrosion system. The effect of two hydrophilic parts and adding a spacer with different functional groups would give valuable information in designing a new Gemini surfactant as a corrosion inhibitor. In 2022, Numin et al. did review the computational simulation studies of Gemini surfactant CI molecules as a corrosion inhibitor. The reviewed manuscript revealed Gemini surfactant CI molecules with a high inhibition efficiency (IE > 90%) consisting of monomer surfactant with quaternary cationic ammonium (N^+^) hydrophilic head, and the typical spacers used are alky, benzene, ester, ether, and ketone. The authors of the review manuscript also found a lack of studies on the acetic acid condition, where corrosion in the presence of acetic acid is one of the biggest corrosion problems faced by oil production pipelines [9]. Therefore, in this study, quantum chemical calculation and molecular dynamic (MD) simulation were used to study the adsorption mechanism of two quaternary ammonium monomer surfactants (2B and H) and their respective Gemini structures with the addition of five different functional group spacers (alkyl, benzene, ester, ether, and ketone) in the acetic acid condition.

## 2. Methodology

### 2.1. DFT Calculation

Quantum chemical methods such as density functional theory (DFT) calculation are used to study the electrochemical processes in the corrosion inhibition mechanism, including the transfer of electrons from the CI molecules to the metal electrode surface [13]. Several parameters extracted from DFT calculation elucidate the adsorption properties of all CI molecules as corrosion inhibitors. The molecular structure of all CI molecules is shown in Table 1. Two quaternary ammonium single surfactants with the presence of naphthalene and hydroxy functional group are named 2B and H, respectively. Then, five spacers with different functional groups (alkyl, benzene ring, ester, ether, and ketone) connect each surfactant molecule to form quaternary ammonium Gemini surfactant molecules (Table 1). TURBUMOLE (Tmolex) software was used to optimize all the CI molecules [14]. The protein data bank file of all molecules is obtained from Automated Topology Builder (ATB) and Repository Version 3.0 [15]. Then, the molecules are optimized using hybrid functional B3LYP [16,17] in a non-aqueous or vacuum environment with def-SV(P).h basis set [14,18]. The ground-state calculation was inserted under the DFT setting to generate the input file. The molecule’s visualization and parameters derived from the DFT calculation were visualized and calculated using the Tmolex program.

The output parameters calculated include the highest occupied molecular orbital energy ($E_{HOMO}$) and the lowest unoccupied molecular orbital energy ($E_{LUMO}$). Then, both HOMO and LUMO energy were used to calculate the other inhibition parameters of ammonium surfactant cationic CI molecules such as band-gap energy (∆E), number of transferred electron (∆N), electronegativity (χ), hardness (η), softness (σ), ionization potential (I) and electron affinity (A). These output-derived parameters, or the global reactivity parameters calculated from the DFT calculation, are commonly used by researchers to explain the reactivity of all CI molecule’s adsorption properties to predict their ability to perform as a corrosion inhibitor [19,20,21] (Global parameters 1, 2 and 3). The HOMO indicates the ability to donate an electron, while LUMO implies the electron-accepting ability of the molecules. The difference between the energy of LUMO and HOMO is the ∆E value (Equation (1)), where the smaller the ∆*E* value, the more reactive the molecules are in accepting and donating electrons.

(1) $$∆E=E_{\mathrm{LUMO}}-E_{\mathrm{HOMO}}$$

According to Koopman’s theorem, the ionization potential (I) and electron affinity (A) of the inhibitors are calculated using the equation as follows [22];

(2) $$I=-E_{\mathrm{HOMO}}$$

(3) $$A={-E}_{\mathrm{LOMO}}$$

Therefore, the electronegativity (χ) and the chemical hardness (η) value that explains the ability to attract electrons and portrays the resistance towards the deformation of electron cloud around the molecules, respectively, can be calculated using the following equations (Equations (4) and (5)), according to Pearson [23];

(4) $$\chi =\frac{I+A}{2}$$

(5) $$\eta =\frac{I-A}{2}$$

Other than that, the other parameter is the global chemical softness (σ) where it can describe the capacity if an atom or groups of atoms to receive electrons, was calculated using the following equation [24]:

(6) $$\sigma =\frac{1}{\eta }=\frac{2}{E_{\mathrm{HOMO}}-E_{\mathrm{LUMO}}}$$

The number of transferred electrons from ammonium surfactant cationic CI molecules to the metal surface was also calculated using the *χ* and *η* value of the CI molecules and the composition of the metal (Equation (7)). The $\chi_{inh}$, $\chi_{Fe}$, $\eta_{inh}$, and $\eta_{Fe}$ indicate the electronegativity of CI, the electronegativity of iron (Fe) metal, the chemical hardness of CI, and the chemical hardness of Fe metal, respectively. The theoretical value of $\chi_{Fe}$ is 7.0 eV, and $\eta_{Fe}$ is 0 eV [25,26].

(7) $$∆N=\frac{\chi_{Fe}-\chi_{inh}}{2(\eta_{Fe}+\eta_{inh})}$$

### 2.2. MD Simulation

MD simulation investigated the adsorption strength, diffusion coefficient, and molecular aggregation of CI molecules in the aqueous systems with acetic acid. All the quaternary ammonium cationic Gemini surfactant CI and all molecules in the corrosion system were simulated using GROMACS software package 4.5 [27]. The corrosion systems include the Fe (110) metal surface, 3% sodium chloride (NaCl), and 1500 ppm acetic acid. The Fe (110) metal surface was used as a metal surface due to its high surface area and high stability compared to other metals [9]. In the adsorption energy calculation, one CI molecule is added into the system, while for diffusion coefficient calculation and molecular aggregation analysis, the CI was added at five different concentrations (0.04 M, 0.08 M, 0.12 M, 0.16 M, 0.2 M).

**Figure 1:**
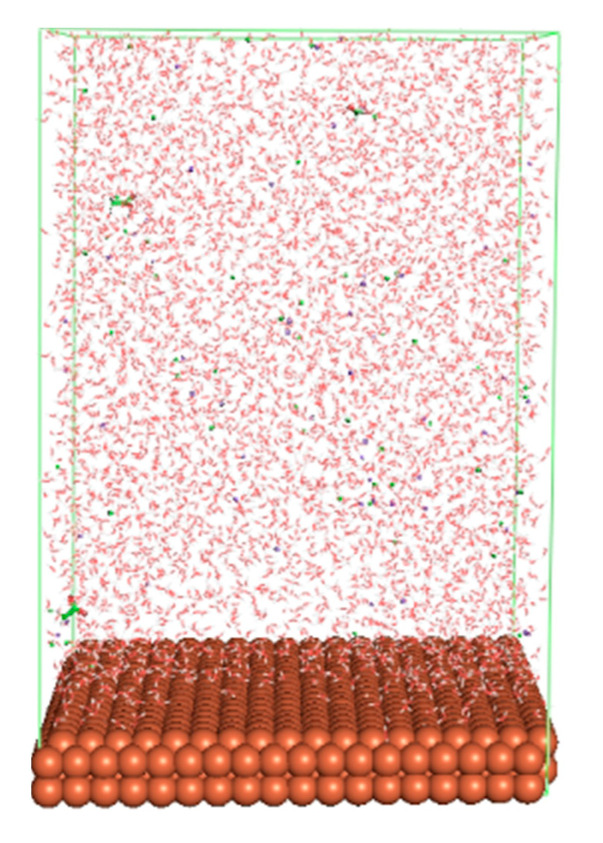
The corrosion system of Fe (110) as a metal in 3% NaCl, and 1500 ppm acetic acid.

The pre-simulation started with the construction of the corrosion system, as shown in Figure 1. The box size used are (51.60, 51.60, 77.40) Å, and the periodic boundary condition (PBC) was applied in all directions (x, y, z) with 2.0 fs time steps. After that, the energy minimization was performed to begin the simulation using the steepest descent followed using a conjugate gradient for 5000 steps. The simulation then proceeded with the 5 ns of NVT to establish the proper orientation in the system’s temperature for the adsorption’s energy calculation. Then, the NPT ensemble was run for 5 ns to analyze the molecular aggregation and calculate the diffusion coefficient of CI molecules in the corrosion particles. Electrostatic interaction was calculated using Particle Mesh Ewald (PME) [28] with a grid spacing of 0.12 nm and fourth-order interpolation. The Coulomb and Lennard interactions were summed up to 1.2 nm. The neighbor searching for 1.2 nm was updated every five steps. The bond lengths for the solute, as well as organic solvent molecules, were constrained with Linear Constraint Solver (LINCS) [29], while for all water molecules, with SETTLE algorithm [30].

## 3. Results and Discussion

### 3.1. Method Validation

Method validation is carried out to validate the computational method used in the analysis by comparing the values calculated from the computational and values from the literatures. It is essential to produce accurate results based on the molecular conditions in each ensemble. In the DFT calculation, acetic acid’s bond length and energy were used to validate the method used to analyze the adsorption properties of the CI molecules. The calculated bond length and energy of optimized acetic acid using DFT calculation and the values from the literatures are tabulated in Table 2. Based on Table 2, the comparison of bond length and total energy of acetic acid calculated from the DFT calculation and literature shows a good agreement with a low percentage error, indicating the accuracy of the DFT method used to analyze the adsorption and electronic properties of the CI molecules. For MD simulation analysis, the calculated density of the water at the corrosion system was compared with the literature to validate the method used for corrosion inhibitor analysis. The density value of the water system calculated from the MD simulation is 860 g/cm^3^, while the value from the literature [31] is 952.3 g/cm^3^. The value of density of the water from the MD simulation method used with literature comparison also proved the accuracy and the ability of the MD simulation method used with a low percentage error (9.69%), indicating that both computational methods used (DFT calculation and MD simulation) can be used to continue investigating the adsorption of the CI molecules towards the metal surface.

### 3.2. DFT Calculation

The inhibitor and metal interactions are mainly caused by the electron donation mechanism from a lone pair at the heteroatom of CI to the empty d orbital of metal. The region or atom that can donate electrons is known as the highest occupied molecular orbital (HOMO). Another interaction is by the acceptance of electrons from the d orbitals of the metals to the empty orbitals of the CI molecule, known as the lowest unoccupied molecular orbital (LUMO). The tendency to donate and accept electrons of the CI molecules can be explained by the determination of HOMO and LUMO energy ($E_{HOMO}$ and $E_{LUMO}$), respectively. The higher the HOMO energy value, the higher the tendency of CI to donate electrons to the vacant orbital of the metal surface, whereas the lower LUMO energy value indicates the ability to accept electrons of the molecules [13,34]. Table 1 and Table 3 show all CI molecules’ optimized structures, HOMO and LUMO. For a single surfactant, 2B, the HOMO and LUMO region focus on the benzene ring of the molecule to represent the reactive region for electron transfer. This is due to the benzene ring’s aromatic properties, consisting of localized π electrons that can enhance the electron transfer mechanism [35]. The formation of Gemini from single 2B molecules with the addition of a different spacer does not affect the HOMO and LUMO region of the molecule, except for 2B-ether, where the ether spacer becomes the LUMO region of the molecule. On the other hand, for a single surfactant molecule H, the carbonyl groups (C=O) are the HOMO and LUMO, indicating this molecule’s reactive region. The presence of 2 lone pairs of electrons at the oxygen atom of the carbonyl group gave the ability to donate electrons to the empty d orbital of metals. The formation of Gemini from this H molecule with the addition of a spacer also does not affect the HOMO and LUMO region, where the carbonyl group is still the reactive region for electron transfer. However, for molecule H-benzene, it is obvious that HOMO and LUMO electronic densities are distributed at the benzene spacer due to the presence of π electrons that stabilize the molecule [36].

The ability of the CI molecules as donor-acceptor contributor is investigated using the HOMO, LUMO energy and the other parameters derived from the energy value. Table 4 shows the HOMO LUMO energy, and the parameters derived to explain the adsorption properties of all CI molecules. Comparing 2B and H surfactant CI, 2B has a better electron donating ability and is more reactive with a higher HOMO energy and low band gap energy value than H. The benzene ring attached to carbon can act as a nucleophile due to the rich electron densities that enhance the electron-donating ability and reactivity of the 2B CI molecule compared with adding a hydroxyl group in the H CI molecule [37]. Then, the adsorption properties of both single surfactants, 2B and H, were compared with their Gemini structures. Among all CI molecules, adding benzene and ester spacers into the 2B molecule increases its reactivity with a lower bandgap energy value for the Gemini molecules compared to others. 2B-benzene shows the most promising adsorption ability with the highest HOMO energy and lowest bandgap energy value, proving the importance of the electron-rich benzene ring as an electron donor region to donate electrons from CI to empty d orbital of metal [38]. A review by Numin et al. (2022) [9], Hassan et al. (2022) [39], and Brycki et al. (2021) [40] also found that the majority of corrosion inhibitor that has a higher inhibition efficiency (>90%) are CI molecules that have an aromatic ring functional group. Thus, the analysis using DFT calculation has predicted that Gemini surfactant CI, 2B-benzene has the highest promising ability to work as a better corrosion inhibitor due to presence of an electron-rich density at the benzene ring region that can be an electron donor or act as a nucleophile to an empty d orbital of metal.

### 3.3. MD Simulation

The MD simulation methods were used to study the strength of adsorption, diffusion coefficient, and molecular aggregation of all CI molecules in the corrosion system.

#### 3.3.1. Adsorption Energy

MD simulation calculated the adsorption energy ($E_{ads}$) between the CI molecules and Fe (110) metal surface to define the strength of adsorption in the presence of 3% NaCl and 1500 ppm acetic acid. The equation used to calculate the $E_{ads}$ is shown below [41].

(8) $$E_{ads}=E_{total}-(E_{surface+solution}+E_{inhibitor})$$

where the $E_{total}$ is the energy for corrosion system with CI molecule, $E_{surface+solution}$ is the energy for corrosion system without CI molecule, and $E_{inhibitor}$ is the energy of the system with CI molecule only. Therefore, in this calculation, three different systems (corrosion system with the presence of CI, corrosion system without CI, and system with CI only) were constructed to find the Eads value.

**Figure 2:**
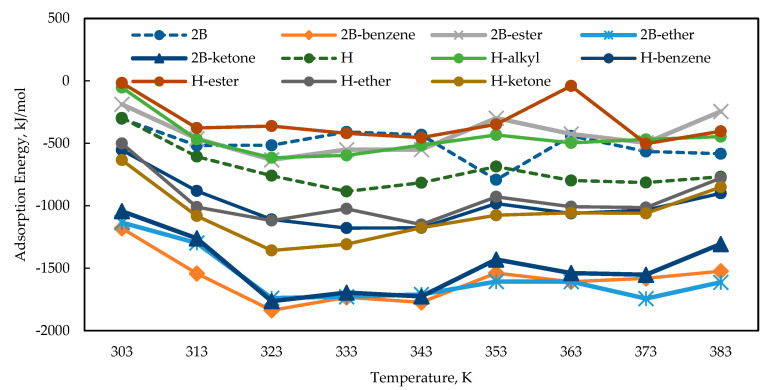
Adsorption energy of all CI molecules in 3% NaCl, and 1500 ppm acetic at temperature 303 to 383 K.

Figure 2 shows the adsorption energy of all CI molecules towards Fe (110) metal surface from temperature 303 to 383 K, with 3% NaCl and 1500 ppm acetic acid. The results show that the Gemini surfactant CI has a higher negative adsorption energy value and represents the higher strength of adsorption compared to its conventional single surfactant except for H-alkyl, H-ester, and 2B-ester. Having two surfactant molecules in one Gemini surfactant structure and adding a spacer with electron-rich heteroatoms increased the adsorption strength of the CI molecules towards Fe (110) metal surface compared to its conventional ones [42]. The adsorption energy calculation results correlate with the DFT calculation, where the 2B-benzene molecules with the most promising electron-donating ability also have the greatest strength of adsorption with the highest negative adsorption energy value. The strength of adsorption is the primary factor influencing the inhibition mechanism of surfactant molecules. The study by Zhu et al. in 2021 supported this statement where the higher adsorption energy value of Gemini surfactant calculated from MD simulation has a higher inhibition efficiency than its conventional monomer with a lower adsorption energy value [41]. The temperature does not affect the strength of adsorption for all CI molecules, indicating a good heat resistance that can adsorb onto the metal surface at extreme temperatures. The unique structure of Gemini surfactant contains a pair of surfactant monomers and a spacer to attach them is well-known for their application in extreme conditions such as high temperatures [40]. Therefore, based on the adsorption energy calculation, Gemini surfactant 2B-benzene is assumed to have a better inhibition efficiency with the highest negative value of adsorption energy.

The bandgap and adsorption energy values of the CI molecules are the critical parameters used for the inhibition efficiency prediction of the CI molecules using DFT calculation and MD simulation, respectively. Studies from the literature have shown that the molecule with a low bandgap energy and high adsorption energy towards the metal surface will experimentally give a higher percentage of inhibition efficiency. Table 5 below shows the bandgap and adsorption energy values of the best CI molecule predicted from this work (2B-Benzene) and the molecules from previous results in the literature. The table shows that the 2B-Benzene CI molecule has a lower bandgap energy value and higher adsorption energy than the molecules from the literature, indicating the ability of the 2B-Benzene to work better as a corrosion inhibitor.

#### 3.3.2. Diffusion Coefficient

**Figure 3:**
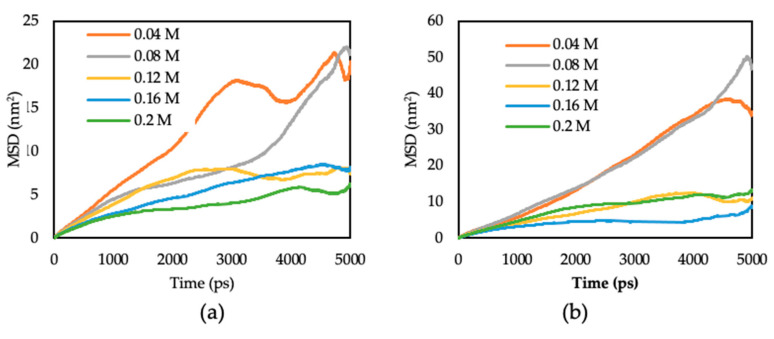
MSD plots of (**a**) 2B and (**b**) H molecules in the corrosion particles at temperature 333 K.

**Figure 4:**
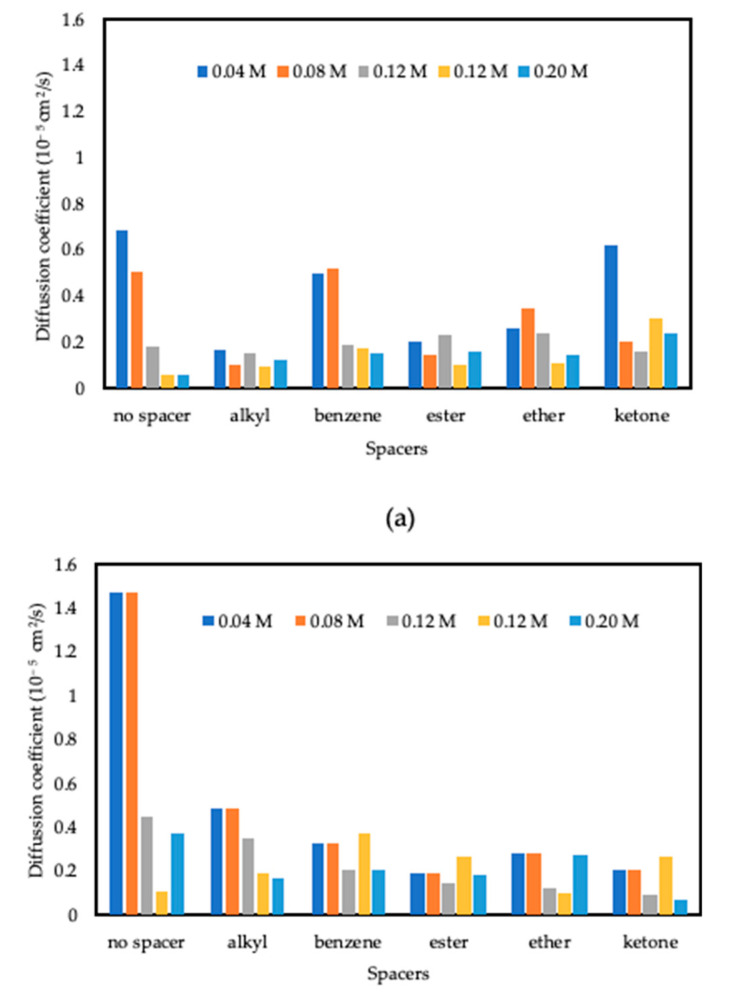
Diffusion coefficient of all CI molecules (**a**) 2B and (**b**) H with their respective Gemini molecules with the addition of different spacers in the corrosion particles at temperature 333 K.

The diffusion coefficient of all CI molecules with the corrosion particles in different concentrations (0.04, 0.08, 0.12, 0.16, and 0.20 M) at temperature 333 K was calculated using the MD simulation method. It is defined as the quantity of a substance diffusing from one region to another, passes through each unit of cross-section per unit of time when the volume-concentration gradient is unit, and can be calculated using the Einstein diffusion Equations below (9) and (10) [43,44]:

(9) $$MSD={\left\{[R_{i}\left(t\right)-R_{i}\left(0\right)\right]}^{2}\}$$

(10) $$D=\frac{1}{6N_{a}}\sum_{i=1}^{N_{a}}{\left\{[R_{i}\left(t\right)-R_{i}\left(0\right)\right]}^{2}\}$$

where t is time, $R_{i}\left(t\right)$ is the position vector of a molecule at time t, and N is the amount of diffusion molecules. The mean square displacement (MSD) derived from the MD simulation for 2B, and H CI molecules is shown in Figure 3. The diffusion coefficient of the molecules is calculated using Equation (10), which represents the limiting slope of the MSD as a function of time, and the values are shown in Figure 4 [45]. Based on Figure 4, all the CI molecules show the same trend where the diffusion coefficient values are high at low concentrations and decrease as the concentration increases. Based on the studies by Hu et al. in 2011, the lower the diffusion coefficient between CI molecules and corrosion particles, the higher the inhibition ability of the CI molecule [45]. The addition of spacers for both surfactant monomers 2B and H gives the same effect trend. The diffusion coefficient for the 2B and H molecules decreases with the addition of spacers at low concentrations (0.04 and 0.08 M). In contrast, at high concentrations (0.12, 0.16 and 0.20 M), the formation of Gemini by adding spacers increases the diffusion coefficient value. This trend shows that higher concentrations of monomer surfactant are equivalent to a lower concentration of its individual Gemini molecules. A unique feature of Gemini surfactant molecules that consist of two surfactant monomers and a spacer gave the ability for the molecules to work better as a corrosion inhibitor with a lower diffusion coefficient at low concentrations compared to its monomer that requires high concentration. The diffusion coefficient analysis proves that Gemini surfactant can inhibit corrosion even at a low concentration compared to its conventional monomers.

#### 3.3.3. Molecular Aggregation

In the molecular aggregation analysis, a system that consists of 3% NaCl, 1500 ppm acetic acid, Fe (110) metal surface, and CI molecules with different concentrations (0.04, 0.08, 0.12, 0.16, and 0.20 M) was constructed. The temperature used in this analysis is 333 K. The behavior of CI molecules was shown in Table 6 and Table 7 to observe the aggregation of the molecules in the corrosion system from low to high concentrations. Table 4 and Table 5 show that both monomer’s surfactant CI molecules are randomly scattered at the aqueous phase of the corrosion system until they reach the highest concentration of 0.2 M, where the molecules start to form a cluster of aggregation. In 2015, Sharma et al. stated that the CI molecules that are randomly scattered tend to have a higher strength of adsorption than in the form of cluster aggregation [46]. The aggregation cluster is similar to the term micelle, where the surfactant forms a cluster of molecules in which the hydrophobic tails are in the interior and the ionic ends on the cluster’s surface. Singh et al. 2019 also found that the unaggregated CI molecules show a strong tendency to adsorb onto metal surfaces than the inhibitor’s micelles [47]. Table 6 and Table 7 show that all CI molecules are forming micelles or cluster aggregation of molecules even at the lowest concentration (0.04 M). This is due to the Gemini surfactant properties that have a lower critical micelle concentration (CMC) value than its conventional one. 0.04 M concentration of the Gemini surfactant CI molecules is equivalent to 22,000 to 35,000 ppm of molecules. Looking back to the definition of corrosion inhibitor, it is a chemical substance that minimizes or prevents corrosion in an environment when added in a minimal amount (ppm) [7], which means that Gemini surfactant with a low CMC value is more suitable to be a promising corrosion inhibitor than its respective monomer. Therefore, the molecular aggregation analysis has revealed the ability of Gemini surfactant CI to be a better corrosion inhibitor at a low concentration with a formation of cluster aggregation at 0.04 M. The results from the diffusion coefficient and molecular aggregation correlate to each other, where it is found that the lower concentration of Gemini CI molecules has a lower diffusion coefficient value and less micelle formation that favored the inhibition. As the concentration increases, the diffusion coefficient value increases because more molecular cluster aggregation or micelles form in the aqueous phase.

## 4. Conclusions

The adsorption properties of monomer CI surfactant and its Gemini molecules with the addition of different spacers are successfully investigated using DFT calculation and MD simulation methods. In DFT calculation, adding a spacer to form Gemini molecules for H CI molecules affects the HOMO and LUMO or reactive region, where it is distributed at the spacer of its Gemini structure. On the other hand, adding a spacer to form Gemini molecules for 2B CI does not affect the HOMO and LUMO region, where it focuses on the electron-rich benzene ring functional group. Among all CI molecules, adding a benzene spacer to the 2B molecule and forming a 2B-benzene gave the best CI adsorption properties with the highest electron-donating ability based on the highest HOMO energy value (−2.879 eV) and the most reactive based on the lowest bandgap energy value (0.765 eV) calculated from DFT calculation. The results from the DFT calculation correlate with the adsorption energy calculated from the MD simulation method, in which 2B-benzene also has the greater adsorption strength with the highest adsorption energy value −1837.33 kJ/mol at temperature 323 K towards Fe (110) metal surface in the 3% NaCl and 1500 ppm acetic acid solutions. The bandgap and adsorption energy calculated from DFT calculation and MD simulation were compared with the existing molecules from the literature, and it has been found that the 2B-Benzene can work as a better CI with a higher adsorption energy and lower bandgap energy values. The literature also supported the results where most CI molecules with the higher inhibition efficiency (>90%) are those with an aromatic ring region. Results also reveal that Gemini surfactant CI is more efficient than its monomers based on the electron-donating ability and strength of adsorption from DFT calculation and MD simulation. The diffusion coefficient calculation and molecular aggregation analysis in the presence of corrosive particles at different CI concentrations were also carried out using MD simulation to understand the CI behavior in the corrosion system. The monomer surfactant CIs (2B and H) requires high concentration to reduce their diffusion coefficient towards the corrosion particles, while their Gemini structure can lower the value at low concentration. At the same time, the molecular aggregation analysis also reveals that a lower concentration of Gemini can form a cluster of aggregation compared to its respective monomers that require more higher concentration. Therefore, the diffusion coefficient and molecular aggregation analysis have uncovered that only a small amount of Gemini surfactant is needed for it to work as a better corrosion inhibitor than its monomer surfactant, which requires high concentrations. An in-depth study on the adsorption properties of surfactant CI molecules using computer modeling can be a valuable reference in developing or designing new CI technologies. Computer modeling is a cost- and time-friendly technique that is beneficial in predicting and screening the various surfactant CI efficiencies before it is developed or synthesized experimentally in the physical laboratory, which will require more time, higher costs, and chemical use with a trial-and-error basis. Thus, it is concluded that the versatile structure of Gemini surfactant, powerful computers and quantum chemistry software analysis and the continuous development of elegant synthesis techniques will deliver a broader Gemini surfactant as an effective corrosion inhibitor in mitigating corrosion, focusing on the oil/gas storage refineries, transportation, pipelines, and acidification processes.

## Figures and Tables

**Table 1:** Optimized structure, HOMO, and LUMO of CI molecules for 2-Benzene (2B).

CI Molecule	Optimized	HOMO	LUMO
2B	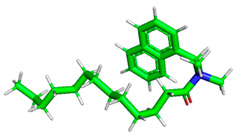	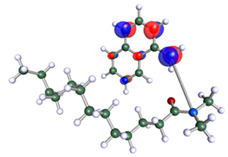	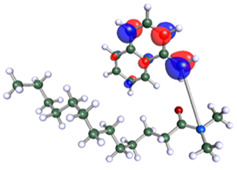
2B-alkyl	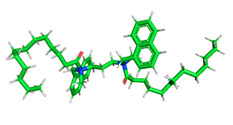	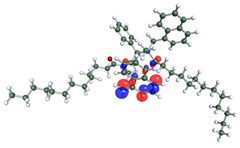	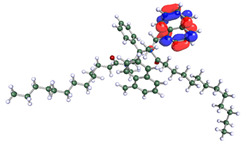
2B-benzene	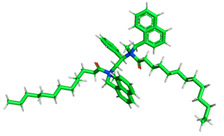	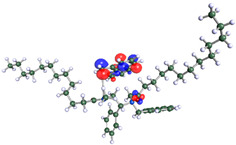	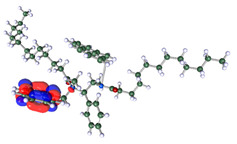
2B-ester	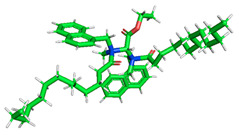	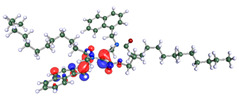	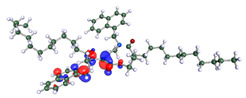
2B-ether	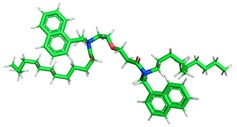	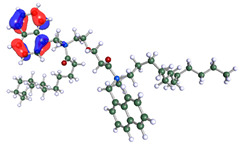	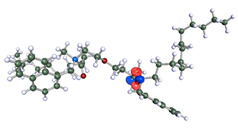
2B-ketone	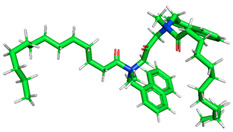	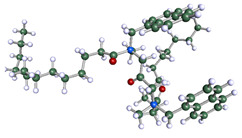	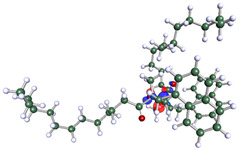

**Table 2:** Bond length and total energy value of acetic acid calculated using DFT calculation and values from literatures.

Bond Lentgh (Å)	Calculated Value	Literature 1 [32]	Percentage Error (%)	Literature 2 [33]	Percentage Error (%)
C1-C2	1.522	1.508	0.066	1.507	0.995
C1-H4	1.099	1.090	0.275	1.093	0.549
C1-H5	1.106	1.095	0.274	1.098	0.729
C1-H3	1.106	1.095	0.274	1.098	0.729
C2=O6	1.198	1.210	3.223	1.249	4.083
C2-O7	1.360	1.359	3.238	1.403	3.065
O7-H8	0.973	0.976	0.922	0.985	1.218
Energy (kJ/mol)	−228.773	−229.082	2.785	−222.701	2.727

**Table 3:** Optimized structure, HOMO, and LUMO of CI molecules for hydroxy (H).

CI Molecule	Optimized	HOMO	LUMO
H	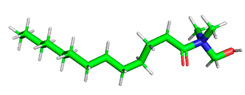	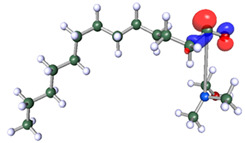	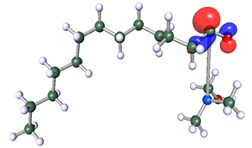
H-alkyl	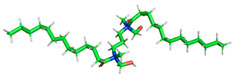	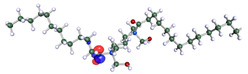	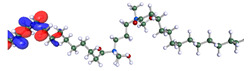
H-benzene	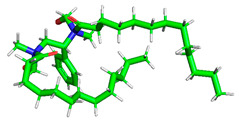	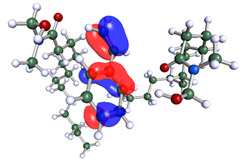	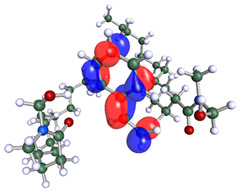
H-ester	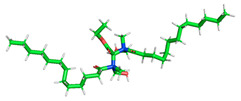	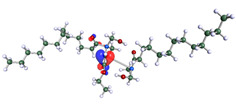	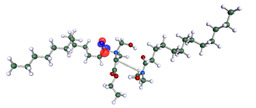
H-ether	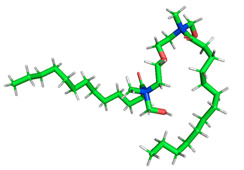	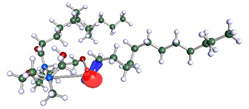	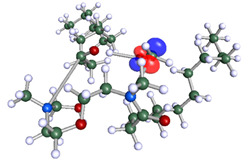
H-ketone	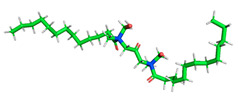	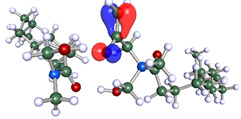	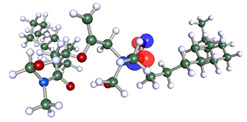

**Table 4:** Derived parameters value from DFT calculation for all CI molecules.

CI Molecule	$E_{\mathrm{HOMO}}$	$E_{\mathrm{LUMO}}$	∆E	Ionization Potential, I	Electron Affinity, A	Global Hardness, η	Electronegativity, χ	Softness, σ	Fraction of Transferred Electron, ∆N
2B	−4.552	−2.079	2.473	4.552	2.079	1.237	3.316	0.809	1.490
2B-alkyl	−2.879	−2.117	3.116	10.729	7.614	1.558	9.171	0.642	−0.697
2B-benzene	−2.879	−2.114	0.765	2.879	2.114	0.382	2.497	2.616	5.890
2B-ester	−4.691	−3.118	1.573	4.691	3.118	0.786	3.905	1.272	1.968
2B-ether	−10.229	−7.124	3.105	10.229	7.124	1.552	8.676	0.644	−0.540
2B-ketone	−10.729	−7.614	3.116	10.729	7.614	1.558	9.171	0.642	−0.697
H	−5.140	−1.570	3.570	5.140	1.570	1.785	3.355	0.560	1.021
H-alkyl	−10.849	−7.665	3.184	10.849	7.665	1.592	9.257	0.395	−0.709
H-benzene	−5.989	−0.920	5.069	5.989	0.920	2.535	3.454	0.395	0.699
H-ester	−5.216	−0.868	4.348	5.216	0.868	2.174	3.042	0.460	0.910
H-ether	−5.083	−1.924	3.159	5.083	1.924	1.580	3.503	0.633	1.107
H-ketone	−4.631	−1.867	2.765	4.631	1.867	1.382	3.249	0.723	1.357

**Table 5:** Bandgap energy, adsorption energy, and inhibition efficiency of 2B-Benzene and CI molecules from literatures.

Molecule	Bandgap Energy (eV)	Adsorption Energy (kJ/mol)	Inhibition Efficiency (%)
2B-Benzene	0.756	−1837.33	-
N,N′-(((1,4-phenylenebis(methylene)) bis(oxy)) bis(ethane-2,1-diyl)) bis (N,N- dimethyldodecan-1-aminium) dibromide [41]	1.62	−1195.54	98.3
4-{[(2E)-3-phenylprop-2-en-1-yl] amino} phenol [43]	3.99	−541.55	96.72
Bis-Mercaptobenzimidazole [44]	3.34	−748.43	92

**Table 6:** Cluster aggregation of all 2B molecules and its respective Gemini molecules at different concentrations.

CI Molecule	Cluster Aggregation				
	0.04 M	0.08 M	0.12 M	0.16 M	0.20 M
2B	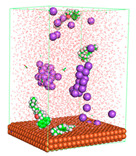	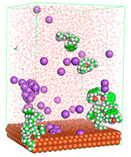	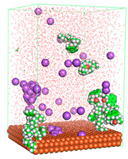	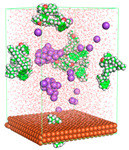	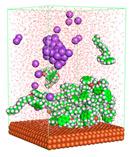
2B-alkyl	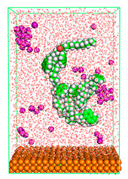	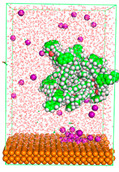	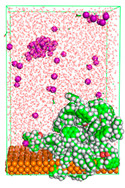	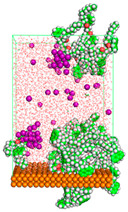	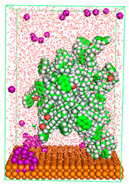
2B-benzene	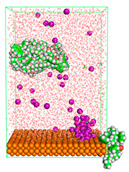	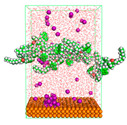	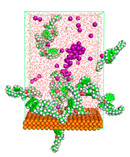	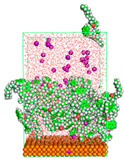	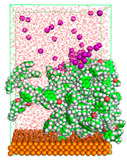
2B-ester	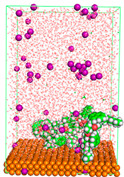	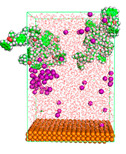	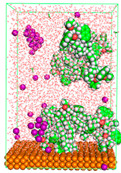	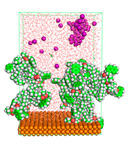	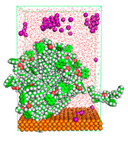
2B-ether	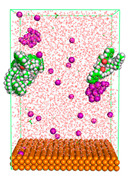	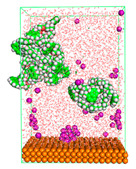	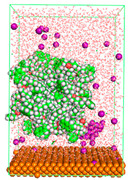	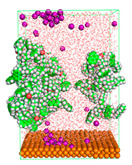	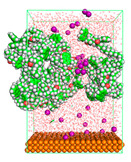
2B-ketone	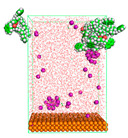	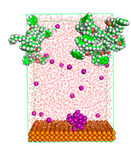	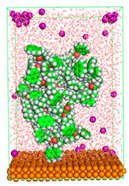	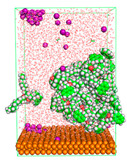	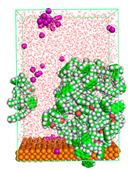

**Table 7:** Cluster aggregation of all H molecule and its respective Gemini molecules at different concentrations.

CI Molecule	Cluster Aggregation				
	0.04 M	0.08 M	0.12 M	0.16 M	0.20 M
H	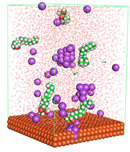	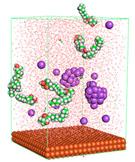	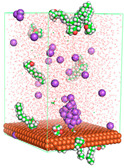	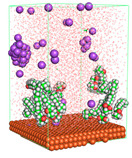	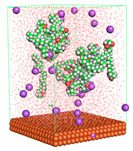
H-alkyl	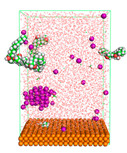	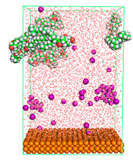	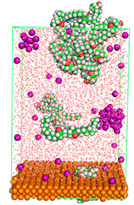	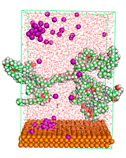	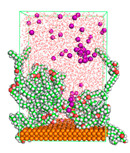
H-benzene	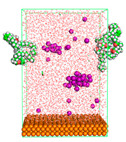	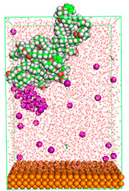	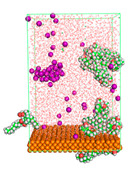	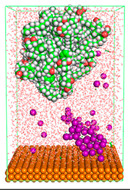	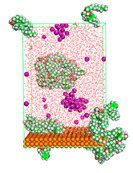
H-ester	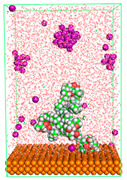	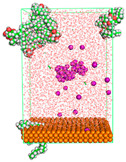	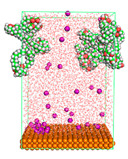	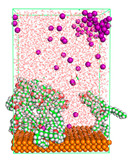	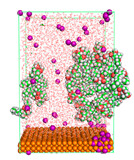
H-ether	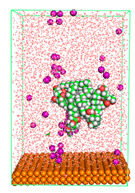	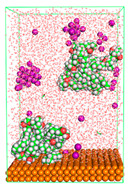	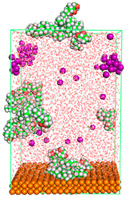	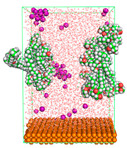	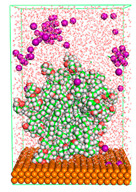
H-ketone	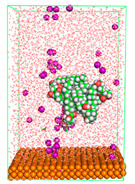	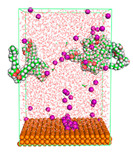	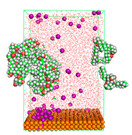	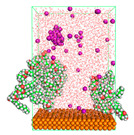	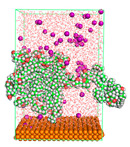

## Data Availability

The data presented in this study are available on request from the corresponding author.
